# The Feasibility of Prehabilitation as Part of the Breast Cancer Treatment Pathway

**DOI:** 10.1002/pmrj.12543

**Published:** 2021-02-10

**Authors:** Fiona Wu, Roberto Laza‐Cagigas, Aalia Pagarkar, Adeola Olaoke, Mohsen El Gammal, Tarannum Rampal

**Affiliations:** ^1^ Surgery Department Medway Maritime Hospital Gillingham UK; ^2^ Prehabilitation Unit Medway Maritime Hospital Gillingham UK

## Abstract

**Background:**

There is compelling support for implementing prehabilitation to optimize perioperative risk factors and to improve postoperative outcomes. However, there is limited evidence studying the application of multimodal prehabilitation for patients with breast cancer.

**Objective:**

To determine the feasibility of multimodal prehabilitation as part of the breast cancer treatment pathway.

**Design:**

This was a prospective, cohort observational study. Breast cancer patients undergoing surgery were recruited. They were assigned to an intervention or control group according to patient preference.

**Setting:**

UK prehabilitation center.

**Participants:**

A total of 75 patients were referred during the study period. Forty eight patients (64%) did not participate; 20 of those opted to be in the control group. Twenty four patients engaged with prehabilitation and returned completed questionnaires. In total, 44 patients were included in the analysis.

**Interventions:**

The program consisted of supervised exercise, nutritional advice, smoking cessation, and psychosocial support.

**Outcome Measures:**

Feasibility was determined by the center's ability to deliver the program. This was measured by the number of patients who wanted to access the service, compared with those able to. Service uptake, patient satisfaction, and project costs were recorded. Patient‐reported outcomes (PROs) and the use of healthcare resources were also evaluated.

**Results:**

A total of 61 patients (81%) wanted to participate; 24 (32%) were able to partake and return questionnaires. Reasons for nonparticipation included surgery within weeks, full‐time commitments, and transportation difficulties. A total of 25 (93%) prehabilitation patients recorded high satisfaction with the program. There was a significant reduction in anxiety among prehabilitation patients. There were no significant improvements in the other PROs. There were no changes to hospital length of stay, readmissions, and complications.

**Conclusions:**

Multimodal prehabilitation is a feasible intervention. Logistical challenges need to be addressed to improve engagement. These results are limited and would require a larger sample to confirm the findings. Work on a thorough cost‐benefit analysis is also required.

## Introduction

Breast cancer remains the most diagnosed cancer among women globally.^1^ Life expectancy is increasing and thus the incidence rates of cancer are also increasing.^2^ For many breast cancer patients, surgical resection of the primary tumor forms an essential part of their treatment with intent to cure. However, surgery is not without challenges. Surgery can have a profound psychological and physiological response on the patient's body and is associated with a decline in functional capacity.^3^, ^4^ It remains a challenge to ensure that these patients are in optimal physical condition before surgery.

Cancer care treatment has traditionally been focused on the postoperative period to rehabilitate and facilitate the patient's recovery.^5^ Multidisciplinary perioperative care programs, such as enhanced recovery after surgery, have been widely implemented and have proven to reduce inpatient hospital length of stay and improve postoperative outcomes.^6^, ^7^ Similarly, early rehabilitation interventions among breast cancer patients have demonstrated improved shoulder mobility and reduced pain, thereby improving quality of life.^8^, ^9^, ^10^

There is a growing focus on developing and implementing interventions earlier in the patient's cancer treatment pathway (immediately following diagnosis).^11^ These interventions provide a continuum of care between diagnosis and the beginning of treatment. They are aimed at optimizing perioperative risk factors to enhance the patient's functional capacity and to mitigate the short‐ and long‐term sequelae of cancer treatment, within the context of an enhanced recovery program.^12^, ^13^, ^14^ The concept of prehabilitation (“prehab”) was established to achieve those goals.^15^, ^16^ Prehabilitation has been shown to improve the physical function of patients preoperatively, thereby improving postoperative outcomes, such as a reduction in the length of hospital inpatient stay, complications, and hospital readmissions.^17^, ^18^ There is a potential saving in healthcare costs resulting from these outcomes.^19^

There is already a multipronged innovation project for colorectal and urology cancer patients underway by the prehab team in February 2018, at a district general hospital situated in Kent (England).^20^ This initiative was modeled on existing prehabilitation programs, whereby there has been an emphasis on improving multiple health behaviors.^21^, ^22^ The project incorporated cross‐health partnerships with the relevant services to optimize premorbid health states, for example, physical exercise, healthy eating, and smoking cessation. It is an interprofessional program allowing for multiple healthcare professionals to coordinate care to facilitate optimal use of the preoperative time and to embed behavioral change before surgery. The prehabilitation innovation project was expanded the following year to include breast cancer patients undergoing surgery.

Currently there are limited published data studying the application of multimodal prehabilitation on patients undergoing breast surgery.^23^ The evidence in support for prehab has been focused on major cancer resections (eg, lung and colorectal cancer).^24^, ^25^, ^26^ There are logistical challenges associated with delivering prehabilitation for breast cancer patients. The waiting time between diagnosis and surgery is often shorter for this cohort of patients owing to the aggressive nature of their treatment.^27^ Furthermore, patients are required to organize time from their schedules to attend prehab at short notice and to arrange for individual hospital transport. The purpose of this pilot study was to assess the feasibility of multimodal prehabilitation as part of the breast cancer treatment pathway. Our pilot study operated over 6 months. The feasibility of the program is reflected in the center's ability to deliver the program.

## Methods

### Study Design

This was a prospective, cohort observational study. We used the basic components of the Health Belief Model regarding behavioral change to support the design of the study.^28^ From October 2019 to March 2020, breast cancer patients requiring surgery were identified and referred for prehabilitation before surgery from a district general hospital. Patients were recruited to the study if they were aged ≥18 years old with a breast cancer diagnosis that was deemed operable by the breast cancer multidisciplinary team and had sufficient knowledge of English to understand and answer the questionnaires. Exclusion criteria were nonsurgical treatments. Prehab was recommended to the patients by their surgeon. The patients opted in or out of the prehabilitation service being offered. They were assigned to an intervention or control group according to their option. The patients consented to participating in the study and completing the questionnaires to be used for the analysis of this study.

### Service Design

Our intervention was modeled on existing prehabilitation programs.^22^, ^29^, ^30^ Unimodal prehabilitation has demonstrated limited impact. Hence, a multimodal approach was adopted.^31^, ^32^ Our program consisted of four key interventions: (1) supervised exercise, (2) nutritional advice, (3) smoking cessation, and (4) psychosocial support. Prehab was introduced at the time of cancer diagnosis. This provided an opportunity to use the waiting time between diagnosis and scheduled surgery to promote healthy lifestyle behaviors. The turnaround time from diagnosis to surgery is short and as such, a limited cohort of patients were admissible to attend a prehabilitation program. Therefore, the intervention group was subdivided and categorized according to the number of supervised exercise training sessions they attended: 0 sessions (control group), 1‐3 sessions, or ≥4 sessions, to determine if a 2‐week program was sufficient to modify lifestyle behaviors and improve patient‐reported outcomes.

### Core Project Team

Our core project team consisted of an anesthetist and clinical exercise physiologists. The primary role of the anesthetist is to be involved with clinical engagement. Anesthetists are uniquely placed to support prehab as they assess and risk stratify patients for their surgical suitability in the preassessment clinic and form perioperative management plans for patients undergoing surgery. Their role is supported by the clinical exercise physiologists who are involved with designing, implementing, and delivering the prehabilitation program.

### Multimodal Prehabilitation Program

The prehabilitation program is a multimodal intervention. Three of the four interventions are advisory and ascribed to one face‐to‐face session (nutrition, smoking cessation, and psychosocial support), whereas exercise involved supervised sessions over the period from diagnosis to operation. Participants were required to attend one or more sessions of supervised exercise. The length of the intervention depended on the length of time to surgery. Each session lasted for 30 minutes and was organized into two sessions per week, until the date of their operation.

#### Supervised Exercise

The supervised exercise sessions are focused on upper body resistance training (RT). RT has been coupled to improve fatigue, muscle strength and shoulder mobility; these limitations are often associated with breast cancer surgery.^33^, ^34^, ^35^ In addition, an increase in physical activity can reduce the risks of noncommunicable diseases and improve both life expectancy and the probability of breast cancer mortality.^36^, ^37^ The RT protocol consisted of two circuits of exercises. The first circuit (sit‐to‐stand, horizontal row, calf raises, and chest press) is repeated three times before proceeding with three repetitions of the second circuit (deadlift, pullover, knee extension, and shoulder press). Each exercise is performed between 8 to 12 times to comprise a total of 24 to 36 repetitions per exercise per session. For each given exercise, the resistance is increased when the patient can perform 36 repetitions with good form during a session. The circuit‐based RT protocol allowed us to accommodate this intervention to more than one participant simultaneously (up to six patients at any one time) and allowed patients to replicate these exercises at home. Aerobic exercise was not chosen as one of our interventions given the limited resources (cost and availability). There were only two exercise bikes available in the prehabilitation unit.

#### Nutritional Advice

An initial screening survey was performed by the physiologist to identify patients with unhealthy eating habits (eg, diets consisting of highly processed foods and salt). The perioperative nutrition score was used to determine if patients needed clinical nutritional intervention. Nutritional education was provided by the physiologists during individual sessions. Patients were advised to consume adequate amounts of protein from plant‐based and/or animal sources to meet their daily requirements (an average daily intake of 64 g of protein for women). Furthermore, they were advised to consider the quality of their protein, to cut down on processed foods, particularly processed red meat. Protein is essential for tissue repair. Protein deficiency is associated with poor wound healing and increased infectious complications.^38^, ^39^ Processed food has a lower nutritional density. The consumption of processed foods is associated with poorer health outcomes and a host of noncommunicable diseases.^40^ There is an increased risk of breast cancer associated with processed red meat intake.^41^ Information provided was reinforced with dietician‐designed patient information leaflets, which was referenced from the NOVA classification for detection of the extent and purpose of industrial food processing, the European Society for Clinical Nutrition and Metabolism (ESPEN) guidelines, and the American consensus regarding protein intake.^42^, ^43^, ^44^ Adherence was verbally confirmed during the sessions via an interview with the exercise physiologist.

#### Smoking Cessation

Patients who were smoking were supported to attend the smoking cessation service. The stop smoking advisors provided these patients with the necessary therapies and advice to help them quit smoking (eg, nicotine‐replacement therapy, patches, gum). Preoperative smoking cessation is valuable and reduces the risk of cardiopulmonary complications, wound infections, and improves tissue healing.^45^

#### Psychosocial Support

Anxiety is highly prevalent among breast cancer patients.^46^ Psychological distress can have a negative effect on surgical patients and is associated with intraoperative abnormal hemodynamic parameters and increased postoperative pain.^47^ By introducing psychological support before surgery as part of prehabilitation, we aim to assuage these potential consequences. Patients identified with raised anxiety and/or depression scores in the screening questionnaires were assigned a counselor (accredited member of the British Association for Counselling and Psychotherapy). Patients had at least one session with their counselor as part of the prehab program. Patients were taught mindfulness techniques by the counselors. Mindfulness is a mental training practice that involves breathing techniques and meditation as a method of coping with anxiety and reducing stress. This has been demonstrated to bring about numerous psychological benefits, including an improvement in psychological symptoms and fatigue.^48^

### Feasibility

The feasibility of the service was determined by the center's ability to deliver the program. This was measured by the number of patients who wanted to access the service, compared with those that were able to do so. In addition, we recorded service uptake to assess acceptance of prehabilitation among breast cancer patients. This was measured by the number of patients opting to participate. Patient satisfaction was recorded with service evaluation forms.

### Patient‐Reported Outcomes

Three questionnaires were distributed to patients pre‐ and postsurgery. Patients completed the preoperative questionnaires at the time of enlisting for surgery (before undergoing prehabilitation). This formed the baseline assessment. The postoperative questionnaires were collected at 6 weeks postsurgery. This allowed patients to return completed forms at their follow‐up appointment for ease of convenience. These questionnaires involved gathering information on (1) global health status by the Short Form 12 (SF‐12) health survey, (2) mental well‐being by the Hospital Anxiety and Depression Scale (HADS), and (3) shoulder function by the Shoulder Pain and Disability Index (SPADI).

1. The SF‐12 is a screening for quality of life and self‐perceived health status.^49^ The questionnaire contains 12 questions and measures different aspects of quality of life and health. The SF‐12 has two subscales: the Mental Component Summary and the Physical Component Summary. Scores for each component range between 0 and 100, with higher scores indicating better quality of life and health.
2. The HADS contains 14 questions split into two subscales: the anxiety component and the depression component.^50^ A greater score (≥8) indicates a probable case of anxiety or depression. The questionnaire was initially developed to be used in hospitals, but studies have confirmed its validity among both hospital and community patients.^51^
3. The SPADI is a validated measure of both shoulder pain and shoulder disability.^52^ This questionnaire contains 13 items: the first five items provide the pain score and the latter eight items provide the disability score. The increasing values indicate the severity of the shoulder pain and increasing difficulties with activities of daily living.

### Use of Healthcare Resources

Length of inpatient hospital stay and hospital readmissions and complications within 30 days for the same condition were registered.

### Statistical Analysis

The responses from the three patient‐reported outcome questionnaires were compared in the surgical breast cancer patients, before and after undergoing surgery, to determine the changes from the baseline assessment. Pre‐ and postsurgery scores were compared between groups using the Kruskal‐Wallis test. Within‐group comparisons were performed using the Wilcoxon signed‐rank test. Data are presented as median (interquartile range). A *P* value <.05 was considered statistically significant. Statistical analysis was performed using SPSS software and Windows Microsoft Excel.

### Ethical Considerations

This service expansion project was registered as an Innovation and Service adaptation with the local Foundation Trust's Quality Improvement and Clinical effectiveness Team. Project approval was granted by the Foundation Trust. Eligible patients were identified and approached about the study during clinic visits. Eligible patients who agreed to participate in prehabilitation provided informed consent and completed presurgery (baseline) questionnaires.

## Results

A total of 44 patients were included in the analysis of this study. Twenty‐four patients participated in our prehabilitation program. Twelve patients attended ≥4 sessions of supervised exercise training sessions and 12 patients attended 1‐3 sessions. Twenty patients declined to participate but were included in the study as the control group. All patients were female. The median age was 63 years old (range, 30‐86 years old). The modal American Society of Anesthesiologists grade was 2. A summary of the patients' characteristics is outlined in Table 1.

**Table 1:** Summary of patients' characteristics

	Declined Prehab (n = 20)	1‐3 Sessions (n = 12)	≥4 Sessions (n = 12)
Age			
>65 years	8	6	7
<65 years	12	6	5
Ethnicity			
Afro‐Caribbean	0	0	1
Asian	0	1	1
White British	20	11	10
BMI			
>35	4	0	1
30‐35	5	4	6
<30	11	8	5
ASA			
Grade 2	13	9	10
Grade 3	7	3	2
Smoking status			
Smoker at baseline	3	0	2

BMI = body mass index; ASA = American Society of Anesthesiologists.

### Intervention Details

The median program duration was four sessions (ie, 2 weeks; range 1‐13 sessions, or 1‐7 weeks). A summary of the interventions offered to the prehabilitation cohort is described in Table 2.

**Table 2:** Summary of interventions offered to the prehabilitation cohort

Perioperative Risk Factors	Summary of Intervention	Summary of Intervention Offered to Patients Attending Prehabilitation (n = 24)	Number of Sessions Attended
Exercise	Resistance exercise training (up to two sessions per week), with a focus on upper limb exercises.	All 24 patients had exercise as an agreed intervention.	Twelve patients attended 1‐3 sessions of exercise; 12 patients attended ≥4 sessions.
Smoking	Onward referral to smoking cessation services.	Two patients were smokers and were referred to smoking cessation services. One has quit smoking for good and one has cut down.	Smoking cessation advice was provided in one session; further contact was made when the patient required further prescriptions for nicotine‐replacement therapy.
Obesity	Advice on diet to avoid processed foods and to consume adequate amounts of protein.	Ten patients with obesity and one morbidly obese patient received advice on healthy eating and weight management. None of the patients required additional oral nutrition supplementation.	Healthy eating advice was provided in one session; adherence was verbally confirmed during each supervised exercise session with the exercise physiologist.
Anxiety/depression	Onward referral for psychological counseling.	Thirteen patients had raised anxiety and depression scores and were referred to counseling. Patients were taught anxiety and stress‐reduction techniques.	One counseling session was provided before surgery.

### Project Feasibility

#### Patient Participation

A total of 61 (81.3%) patients wanted to participate in the prehabilitation program. Over the 6‐month pilot period, 75 patients were invited to attend the program. A total of 14 (18.7%) patients declined to participate because of a lack of interest. Of 61 (81.3%) patients who wanted to attend prehabilitation, only 24 (32%) patients achieved this and returned results. Three patients completed the program but did not complete the questionnaires. The most common reasons for nonparticipation among those who wanted to attend were surgery within 2 weeks (n = 14); full‐time commitments, for example, work/caring responsibilities (n = 12); and transportation difficulties (n = 8). A total of 20 nonparticipating patients consented to participate in our study and were enrolled in the control group.

#### Patient Satisfaction

Service evaluation forms were completed postoperatively by the 27 prehabilitation patients (inclusive of the three patients who did not return their questionnaires). A total of 25 (93%) patients regarded the service as “excellent” and a powerful motivator for achieving healthy lifestyle changes. They perceived the service to be beneficial, as part of their cancer treatment, their postoperative recovery, and they would strongly recommend it to other patients. Two (7%) patients regarded the service as “average” and subsequently discontinued prehabilitation, citing muscle soreness (n = 1) and lack of interest (n = 1) as reasons for discontinuation.

#### Project Costs

An Independent Cancer Taskforce Strategy funded a £100 000 grant to set up our prehabilitation unit including exercise equipment, patient‐information materials, and staff fees (project manager and physiologists). The prehabilitation unit served colorectal and urology in addition to breast patients from a single district general hospital. The cost of the intervention was £200 to £500 per patient for all three surgical specialties; the cost was based on the number of sessions attended, which was based on the waiting time to surgery.^19^, ^20^, ^22^

### Effect of Prehabilitation on Patient‐Reported Outcomes

**Figure 1:**
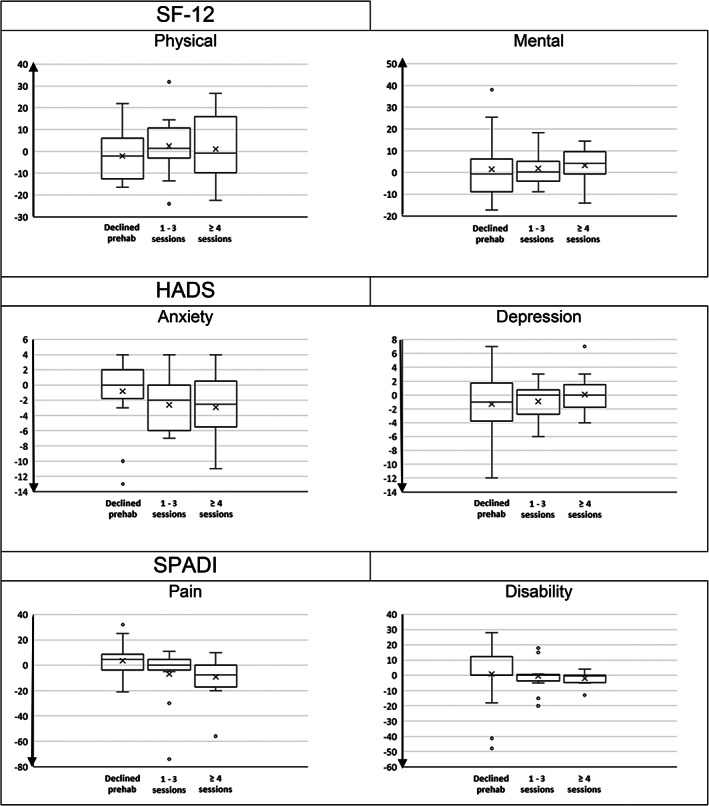
Boxplots of differences between scores at baseline and at 6 weeks postsurgery for the three groups of patients. The arrowhead indicates direction of improvement in scores. HADS = Hospital Anxiety and Depression Score; SF‐12 = Short Form 12; SPADI = Shoulder Pain and Disability Index.

Pre‐ and postsurgery patient‐reported outcome questionnaires were collected to assess the patients' general health status, mental well‐being, and shoulder pain/disability in the SF‐12, HADS, and SPADI, respectively. Pre‐ and postsurgery scores obtained by all three cohorts of patients are outlined in Table 3. Figure 1 shows the boxplots of differences between scores at baseline and at 6 weeks postsurgery for the three groups of patients.

**Table 3:** Patients' scores at baseline and 6 weeks postsurgery

Group	Questionnaire	Component	Baseline	6 Weeks Postsurgery	Z	*P*
Declined prehab (n = 20)	SF‐12	Physical	47.5 [35.4‐55.5]	44.4 [34.9‐52.3]	−0.933	.351
		Mental	47.1 [35.5‐58.9]	54.4 [32.0‐58.2]	−0.149	.881
	HADS	Anxiety	7.0 [3.0‐12.8]	5.0 [1.3‐11.5]	−0.078	.938
		Depression	3.0 [1.0‐9.8]	2.5 [1.0‐8.0]	−1.158	.247
	SPADI	Pain	0.0 [0.0‐12.8]	8.0 [1.0‐17.8]	−1.208	.227
		Disability	0.5 [0.0‐17.5]	8.0 [0.0‐23.5]	−0.589	.556
1–3 sessions (n = 12)	SF‐12	Physical	46.0 [36.9‐51.4]	47.9 [38.5‐53.3]	−0.628	.530
		Mental	53.4 [44.9‐57.1]	51.7 [46.1‐57.6]	−0.628	.530
	HADS	Anxiety	5.0 [1.3‐8.0]	2.0 [1.0‐5.0]	−2.201	.028
		Depression	2.0 [1.0‐6.8]	2.5 [1.0‐3.8]	−1.054	.292
	SPADI	Pain	0.0 [0.0‐16.3]	0.0 [0.0‐4.5]	−0.254	.799
		Disability	0.0 [0.0‐16.3]	0.0 [0.0‐4.0]	−0.210	.833
≥4 sessions (n = 12)	SF‐12	Physical	51.5 [34.6‐55.7]	50.9 [42.2‐54.2]	−0.549	.583
		Mental	51.6 [42.4‐57.4]	56.7 [45.3‐59.0]	−1.491	.136
	HADS	Anxiety	6.5 [1.5‐10.3]	4.0 [2.3‐5.0]	−2.007	.045
		Depression	1.0 [0.3‐4.8]	1.5 [0.3‐3.8]	−0.085	.932
	SPADI	Pain	13.0 [0.0‐27.0]	5.0 [0.0‐9.8]	−1.601	.109
		Disability	1.0 [0.3‐11.0]	0.5 [0.0‐4.8]	−1.409	.159

HADS = hospital anxiety and depression scale; SF‐12 = short form 12; SPADI = shoulder pain and disability index; *P* = significance; Z = *z*‐score.

Values are expressed as median [interquartile range]. Wilcoxon signed‐rank test was calculated to compare the before and after changes.

The between‐group differences in the scores before surgery were not statistically significant. Postsurgery differences between groups did not achieve statistical significance either. When analyzing within‐group pre‐ and postsurgery changes, anxiety scores were statistically lower after surgery in both groups participating in prehabilitation. The remaining components analyzed did not demonstrate significant changes.

### Use of Healthcare Resources

The median length of hospital stay was 2 days/1 night for both the prehabilitation and non‐prehabilitation cohorts. There were no 30‐day complications requiring further hospitalization and no hospital readmissions for the same condition identified in the patients studied.

## Discussion

This pilot study has demonstrated that multimodal prehabilitation for breast cancer patients can be delivered in a district general hospital. A total of 75 patients were referred to prehabilitation. Fourteen (18.7%) patients declined to participate because of a lack of interest and 61 (81.3%) patients wanted to participate. Of those 61 patients, only 24 (32%) were able to achieve this and return completed questionnaires. Although the center had availability to meet 100% of participants’ requirements, uptake was limited from 61 to 24 patients owing to the service being delivered at specific scheduled appointments and at only one location. Work is underway to open satellite community centers and “virtual” prehabilitation to improve patient engagement.

The patient‐reported outcomes suggest that prehab for patients undergoing breast surgery may not have the same full perceived benefits as compared with those undergoing other forms of major surgery.^18^, ^26^ Our intervention identified a significant improvement in the anxiety component of the HADS only. The results did not show significant improvements in the remaining questionnaires. In addition, the traditional markers of perioperative care, for example, length of hospital stay and 30‐day complications requiring readmission or further hospitalization, remained the same. Compared to the potential savings accrued following other forms of major cancer resections, there is no clear demonstrable cost benefit in support of prehabilitation for breast cancer patients.^19^ A factor to consider regarding the lack of difference observed in the patient‐reported outcomes between the intervention and control groups are the patients' characteristics. Breast cancer patients are often younger, generally healthier, and have fewer comorbidities than colorectal cancer patients.^1^, ^53^ Furthermore, the surgical stress response that occurs following other forms of major cancer surgery is greater than for breast surgery; as a result our breast cancer patients may not report the same perceived physical benefits of prehabilitation than other oncological surgical patients.^3^ The effectiveness of prehab for breast cancer patients needs to be proven in a larger‐scale study. A modification and improvement in their lifestyle behaviors in the long term may provide a saving in costs to society, for example, a quicker return to employment and a saving in future healthcare usage costs. The measure of the impact of well‐being factors is beyond the scope of this study.

The final factor to consider is that the interventional period is relatively short. Many of the prehabilitation programs in the literature occur over a longer period, a minimum of 4 weeks.^19^, ^26^ In this study, the mean number of sessions was four (ie, 2 weeks of prehabilitation). The turnaround time between cancer diagnosis and surgery is shorter than other cancer treatment target times, because of the aggressive nature of breast cancer treatment. This was a common reason for patients declining to participate in our intervention; 14 (19%) patients had scheduled operations within 2 weeks. In addition, the short intervention period may have been a barrier in capturing true changes among our prehabilitation patients. Nevertheless, the prehabilitation program used the waiting list times and served as an important checkpoint to supporting the patients while waiting for surgery. It was imperative for the prehabilitation team to counsel patients at the time of diagnosis to maximize that crucial time between diagnosis and surgery to optimize patients. Prehabilitation had no impact on the scheduling of their surgery. Based on these cancer treatment targets and the observations from this study, we have made recommendations for patients to commit to a minimum of 2 weeks of sessions (ie, four sessions) to gain the full benefits.

### Limitations of the Study

The study was based on a limited number of patients. Their breast cancer treatment including type of surgery, adjuvant/neoadjuvant chemotherapy and/or radiotherapy were not recorded. Patients were not compared to a matched cohort. Patients volunteered into the intervention group providing potential for self‐selection bias. More patients with an American Society of Anesthesiologists classification of 3 declined to participate. The significance of this is unknown with the current data we have but an interesting issue to explore. It is not possible to determine if patents who declined the program would have received greater benefit from prehabilitation. Patient outcomes were also collected via self‐assessment and we could not verify adherence to our interventional recommendations. A functional assessment was not conducted because of the extremely short period of time from joining prehab to surgery. The duration of the intervention was not standardized as we accommodated the intervention in the time between referrals and operation date. We do not have long‐term follow‐up data on the number of patients who have continued to follow our recommendations.

Furthermore, patient uptake was limited by time and location availability. The cost benefit of face‐to‐face supervised exercise sessions could be superseded in favor of home‐based or “virtual” programs. Subsequent prehab sessions could be held not only within a hospital setting but also in small groups within the community or even at home via teleconsultations (eg, prerecorded YouTube videos, Zoom). This has the potential to increase the availability of sessions and user acceptance and reduce costs.

### Clinical Implications

Within our sample, there is a high interest (81.3%) among patients to participate. This program is simple to perform and shows potential to be replicated across other breast cancer care units. There was a high level of patient satisfaction with our program; however, uptake was low owing to the rigidity of timing and location of service.

## Conclusion

Prehabilitation services have already been established in many units to serve patients undergoing major cancer resections.^11^, ^22^ To the authors' knowledge, this is the first study to explore the effects of multimodal prehabilitation for patients undergoing breast cancer surgery. Our pilot study shows that patients would overwhelmingly like to participate in this program (81.3%) as part of their breast cancer treatment. This program is feasible and can be delivered. However, accessibility needs serious consideration in respect to the acceptance figures observed and technology solutions should be considered. The program necessitates careful orchestration from a multidisciplinary team to be able to coordinate care and align with other health services, although this did not present issues in the project. Finally, a larger study is required to corroborate the positive results presented here and potentially find further significant benefits with a thorough cost‐benefit analysis.
